# A Case Report of an Atypical Presentation of IgG4-Related Disease and Idiopathic CD4 Lymphocytopenia

**DOI:** 10.1155/2015/512370

**Published:** 2015-09-28

**Authors:** Francesco Rapisarda, Luca Zanoli, Grazia Portale, Salvo Scuto, Pietro Castellino

**Affiliations:** Section of Nephrology, Department of Internal Medicine, University of Catania, 95100 Catania, Italy

## Abstract

The IgG4-related disease is a fibroinflammatory disease characterized by tumefactive lesions, a dense lymphoplasmacytic infiltrate rich in IgG4-positive plasma cells, storiform fibrosis, and, often but not always, elevated serum levels of IgG4. Idiopathic CD4 lymphocytopenia is a heterogenic and rare syndrome characterized by the detection of a persistent absolute CD4 T cells count <300 cells/mm^3^ (or <20% of total T cells) in more than one occasion and no evidence of HIV infection in absence of immunodeficiency or therapy associated with depressed levels of CD4 T cells. We report the case of a 50-year-old man with a multiorgan IgG4-related disease presenting in a temporal association with a profound and symptomatic idiopathic CD4 lymphocytopenia. Both clinical pictures improved after steroid treatment. Idiopathic CD4 lymphocytopenia has been associated with a number of autoimmune conditions but, to the best of our knowledge, this is the first case in which an association with the IgG4-related disease is reported.

## 1. Introduction

The IgG4-related disease is a fibroinflammatory disease characterized by tumefactive lesions, a dense lymphoplasmacytic infiltrate rich in IgG4-positive plasma cells, storiform fibrosis, and often, but not always, elevated levels of IgG4 [1, 2].

The IgG4-related disease has been described in several organs and tissues: pancreas, biliary tract, salivary glands, periorbital tissue, kidneys, lungs, lymph nodes, meninges, aorta, breast, prostate, thyroid, pericardium, and skin [1, 3]. Histopathological characteristics are similar in the different organs concerned with this disease [4–7]. The pathogenesis of IgG4-related disease is not completely understood; evidences point to both autoimmune and allergic disorders. IgG4 are considered to have a role in tolerance to allergens and in responses to various infections, but their physiologic role is still poorly understood. There are emerging data showing that the IgG4 antibodies in this disease represent a downregulatory response to an unknown primary inflammatory stimulus [8]. Immune-mediated mechanisms may contribute to the inflammatory process of IgG4-related disease. Various genetic conditions have been associated with the disease (HLA serotypes DRB1*∗*0405 and DRB1*∗*0401 increase the susceptibility in Japanese population, whereas DQB1-57 mutations are associated with disease relapse in Korean population). Bacterial infections with a molecular mimicry mechanism and autoimmunity are all considered potential initiating mechanisms.

Autoimmunity is widely regarded as the initial immunologic stimulus for the type 2 helper T (Th2) cell immune response that is predominant in IgG4-related diseases. Serum IgG4 from patients with IgG4-related disease binds to the normal epithelia of the pancreatic ducts, bile ducts, and salivary ducts. Potential autoantigens at these sites include carbonic anhydrases, lactoferrin, pancreatic secretory trypsin inhibitor, and trypsinogens. However, these autoantibodies are neither specific for IgG4-related disease nor known to be of the IgG4 subclass. Thus, their role in IgG4-related disease is unclear. Autoimmunity and infectious agents may act as potential immunological triggers of the disease [1]. The predominant immune response is Th2, followed by activation of regulatory T (Treg) cells. This characteristic is at variance with what is observed in the other autoimmune diseases in which the function of Treg cells is impaired [9]. The activation of Treg cells in IgG4-related disease is suggested by the high expression of the forkhead box P3 (FOXP3) mRNA in tissue by the infiltrates of CD4+CD25+ Treg cells at affected sites and by the increased number of CD4+CD25+ Treg cells in the blood. In addition, interleukin-10, which is produced by Treg cells as well as by Th2 lymphocytes and transforming growth factor *β* (TGF*β*), appears to be overexpressed in IgG4-related disease. TGF*β* may play a central role in the fibrosis process [10]. Various interleukins, including IL-4, IL-5, IL-10, and IL-13, are overexpressed in target organs [1, 2, 7, 9]. IgG4 and IgE production is enhanced by the Th2 cells response. Th2-related cytokines such as IL-4 and IL-13 enhance the production of both IgG4 and IgE. In contrast, interleukin-10, interleukin-12, and interleukin-21 shift the balance between IgG4 and IgE, favouring IgG4 production. This is consistent with the hypothesis that the production of IgG4 is enhanced preferentially in the setting of a Th2 cell dominant immune reaction. This selective IgG4 induction is referred to as the modified Th2 response [1–9]. In this paper we reported the association between a rare disease, the IgG4-related disease, and the idiopathic CD4 lymphocytopenia (ICL), a heterogenic and rare syndrome of unknown etiology characterized by the detection of a persistent absolute CD4 T cells count <300 cells/mm^3^ (or <20% of total T cells) in more than one occasion and no evidence of HIV infection in absence of immunodeficiency or therapy associated with depressed levels of CD4 T cells [11]. Idiopathic CD4+ T cell lymphocytopenia is a disorder of unknown etiology and it is viewed as a syndrome that likely encompasses different disorders that have in common the reduction of CD4 cell number. There is evidence for increased activation and turnover of CD4 T cells in patients with ICL. Lee et al. [12] found increased levels of serum lipopolysaccharide (LPS) and markers of CD4+ lymphocyte activation in patients with ICL. Apoptosis of CD4+ lymphocyte may be associated with enhanced expression of Fas and Fas ligand. Various other immune disorders have been described in ICL patients, including low CD8+ T cells counts and defective expression of CXCR4 on the surface of CD4+ cells. The alpha/beta and gamma/delta T cell repertoires of ICL patients are highly restricted which may suggest a defect in maturation or differentiation during T cell development and altered tyrosine kinase activity of p56. ICL has been associated with increases in immature or transitional B cells and increased serum of IL-7 [13].

ICL has been associated with a number of autoimmune conditions [11] but, to the best of our knowledge, this is the first case in which it is reported in patients with IgG4-related disease.

## 2. Case Report

On August 2007 a 43-year-old patient presented with the sudden onset of cholestatic jaundice and the radiologic detection of two pancreatic masses, diffuse lymphadenopathy, and hepatomegaly. To exclude the presence of a lymphoma, the patient underwent exploratory laparotomy. A biopsy of liver and of a lymph node at the level of hepatic hilum and cholecystectomy were performed. Biopsy of pancreatic mass was attended but, for technical reasons, not successful. Histology showed the presence of chronic cholecystitis with hypertrophic reactive lymphadenitis, fibrosis of the cystic duct with strong intramural inflammatory infiltrate, signs of portal fibrosis, and cholestasis in the absence of alterations of the biliary tract. Consequently, a bile duct-duodenal anastomosis was packed in absence of a conclusive diagnosis.

On March 2008, the patient presented a complex clinical picture including an interstitial pneumonitis by* Mycoplasma pneumoniae* and* Chlamydia pneumoniae*, Epstein Barr and cytomegalovirus infections, tubulointerstitial nephritis, severe lymphopenia, and marked reduction of CD4 (214 cells/*μ*L, Tables 1 and 2). CT scan showed the presence of three pulmonary nodules (<1 cm), diffuse lymph node enlargement, and enlarged kidneys and spleen. Laboratory showed increased levels of ESR and CRP, hypergammaglobulinemia, positive reuma test and Waaler Rose tests, presence of serum and urinary kappa and lambda light chains, increased beta_2_ microglobulin, and circulating C1q immunocomplexes. ANA, anti-mitochondrial antibody, anti-bile canaliculi, anti-liver kidney mouse, liver western blot, anti-smooth muscle, pANCA, cANCA, anti-thyroglobulin, and anti-peroxidase were negative. Tests for leishmaniasis, toxoplasma, and HIV were also negative. The bone marrow aspirate excluded lymphoproliferative and parasitic disorders. Renal biopsy showed a tubulointerstitial nephritis with diffuse infiltrate of lymphocytes and plasma cells. Arteries and arterioles were undamaged. Immunohistochemistry revealed the presence of CD20, CD3, and CD68. Immunostaining for kappa and lambda light chains documented a polytypic plasma cells population. Direct immunofluorescence was negative for IgA, IgG, IgM, C3, C1q, fibrinogen, kappa, and lambda light chains. Subject was treated with glucocorticoids with improvement of the symptoms and lymphopenia.

Three years later, a reduction of the number of the CD4 lymphocytes was once again reported (146/*μ*L, CD4/CD8 ratio equal to 0.8) in conjunction with the onset of fatigue, weight loss, night sweats, arthralgia, hemorrhagic rhinitis, multiple caries, and consequent extraction of 10 teeth. After the exclusion of HIV and malignancies (biopsy of an inguinal lymph node, bone marrow biopsy, and PET), an idiopathic CD4 lymphocytopenia was diagnosed. Nevertheless, only a part of the signs was explained by CD4 lymphocytopenia.

On January 2014, there was the first visit to our outpatient clinic for worsening renal function, hypocomplementemia, and polyclonal increase of IgG. CD4 lymphocytes were in the normal range.

The medical history of obstructive jaundice pancreatic mass and interstitial nephritis, together with a marked elevation of IgG4, was suggestive of a IgG4-related disease. At the time of our observation, ultrasound examinations showed hepatomegaly, increased volume of the kidneys with a mild dilatation of chalices and renal pelvis without signs of urinary tract obstructions, and bilateral enlargement of the submandibular glands. IgG4 were mildly elevated (185 mg/dL), ANA were negatives, and CD4 were in the normal range.

After 3 months of prednisone 50 mg/day, we observed an improvement of the renal function paired with a reduction of inflammation, IgG, and kidneys volume and the disappearance of the dilatation of chalices and renal pelvis.

At the time of the submission of the present case report, the patient, treated with prednisone 5 mg/day, is in remission with stable GFR and normalization of IgG4 and CD4 lymphocytes.

## 3. Discussion

The involvement of liver (cholestatic jaundice), pancreas (pancreatic masses and lymphadenopathy) and, subsequently, the respiratory system (interstitial pneumonia, lung nodules, and hemorrhagic rhinitis), urinary tract (tubulointerstitial nephritis with polyclonal infiltration of lymphocytes and plasma cells, bilateral hydronephrosis), and salivary glands (bilateral enlargement of the submandibular glands, multiple caries, and consequent teeth extraction) as well as the detection of hypocomplementemia, increased polyclonal IgG and IgG4 subclass, and the rapid improvement of symptoms and laboratory data after steroid therapy are indicative of IgG4-related disease. On the other hand, the history of opportunistic infections such as CMV, EBV,* Mycoplasma*, and* Chlamydia*, in absence of HIV and malignancies, is the consequence of the idiopathic CD4 lymphocytopenia. Only when considered together, the IgG4-related disease and the idiopathic CD4 lymphocytopenia explain the clinical presentation of the subject.

The HISORT criteria [14] reach a high sensitivity (92%) and specificity (97%) to select subjects with IgG4-related disease. These criteria are based on serologic, radiologic, histopathological, and clinical criteria, including the detection of the type 1 autoimmune pancreatitis, the most common sign of the IgG4-related disease, and the responsiveness to the steroids therapy. In the case reported in this paper, all of these criteria were satisfied with the notable exception of the histologic finding of the pancreas which might have confirmed the autoimmune nature of the disease.

The renal involvement reported in IgG4-related disease is a tubulointerstitial nephritis with polyclonal infiltration of lymphocytes and plasma cells, a membranous nephropathy, and a calicopelvic dilatation secondary to retroperitoneal fibrosis [15–19]. Raissian et al. [15] diagnostic criteria for a IgG4-related tubulointerstitial nephritis include (a) elevated serum total IgG or IgG4 levels (>135 mg/dL); (b) plasma cell-rich tubulointerstitial nephropathy with an absolute number of IgG4 plasma cells >10/HPF, obliterative phlebitis, and storiform fibrosis; (c) increased kidney volume with wedge-shaped or patchy lesions; and (d) demonstration of a IgG4-related disease in other organs. All of these criteria were satisfied, except for the presence of IgG4 at renal biopsy which was not evaluated at time of renal biopsy. In this case, hypocomplementemia was present and it is reported in 70% of patients with IgG4-related disease. In contrast to the other subclasses, IgG4 has low affinity for Fc receptors and C1q and thus minimal ability to activate cells or initiate complement activation and has been traditionally considered to play only a limited role in immune activation. Nevertheless, in some circumstances, IgG4 can act as a rheumatoid-factor and binds the Fc portion of other IgG antibodies, in particular IgG4. This interaction occurs between Fc constant domains and may contribute to the molecule's anti-inflammatory function. Complements levels are a helpful indicator of disease activity in patients with a IgG4-RD and renal disease [1, 16].

The simultaneous presence of a tubulointerstitial nephritis and hypocomplementemia is a laboratory feature in IgG4-related tubulointerstitial nephritis [20, 21]. What is unique in this case is the description of an association of the IgG4-related disease with the idiopathic CD4 lymphocytopenia.

The ICL is commonly associated with several autoimmune diseases such as lupus erythematosus systemic, anti-phospholipid antibody syndrome, Sjogren's syndrome, polyarthritis/vasculitis, psoriasis, lichen planus, autoimmune vitiligo, Behcet syndrome, and Hashimoto thyroiditis as well as the atopic dermatitis, sarcoidosis, and idiopathic obliterative bronchiolitis [11]. However, to the best of our knowledge, this is the first case in which an association between the IgG4-related disease and the ICL is reported. However, the pathophysiology of both the IgG4-related disease and idiopathic CD4 lymphocytopenia is not completely clear. Therefore, it is not possible to confirm the common pathway between these diseases and, inversely, the casualty cannot be excluded. Consequently, it may be of interest to investigate further the temporal relationship of the IgG4-related disease and idiopathic CD4 lymphocytopenia and the exact nature of correlation of the two diagnoses.

## 4. Methodological Issues

The present case report describes the clinical history and follow-up of a IgG4-related disease presenting in association with an apparently idiopathic profound and recurrent decline in CD4+ cells. The major limitation of this study is that, despite the fact that both the HISORT [14] and Raissian criteria [15] required the bioptic demonstration of IgG4, these data were unavailable in our case.

## 5. Conclusion

In conclusion, we reported a case of a subject with a multiorgan IgG4-related disease presenting in a temporal association with a profound and symptomatic idiopathic CD4 lymphocytopenia. Both clinical pictures improved after steroid treatment.

## Figures and Tables

**Table 1:** Main laboratory data.

	2008 [after steroids administration]	2013	2014 [before steroids administration]	2014 [1 month after steroids administration]	2014 [3 months after steroids administration]
Serum creatinine, mg/dL	1.40	1.80	2.08	1.60	1.52
GFR, mL/min/1.73 m^2^	59	43	36	49	52
Proteinuria, mg/24 h	924	342	350		200
ANA		2.9	neg		
Anti-dsDNA		neg	neg		
pANCA		neg	neg		
cANCA		neg	neg		
C3, mg/dL		7.3	63	67	
C4, mg/dL		2.9	3	6	
Circulating immune complexes (*μ*g equiv./mL)	>100				
Rheumatoid-factor, UI/mL [normal <20 UI/mL]	80		217	24	
ESR, mm/h			117	35	
Glycosuria, mg/24 h	3540				
Total IgG, mg/dL	3480		4260	1900	955
IgG1, mg/dL	2670		1470	1190	554
IgG2, mg/dL	821		429	316	247
IgG3, mg/dL	100		126	48	24
IgG4, mg/dL	1380		185	185	185

GFR, glomerular filtration rate.

**Table 2:** Lymphocyte typing of March 2008, before steroids administration.

	Cells/*µ*L		%	
	Measured	Normal range	Measured	Normal range
March 2008				
Total T lymphocyte	472	690–2540	54	55–84
T suppressor lymphocyte	213	190–1140	25	13–41
T helper CD4+ lymphocyte	214	410–1590	25	31–60
NK CD16+ lymphocyte	359	90–590	40	5–27
B lymphocyte	35	90–660	4	6–25
CD8+CD38+CD3 lymphocyte			18	2–5
January 2014				
Total T lymphocyte	2058	690–2540	58	55–84
T suppressor lymphocyte	478	190–1140	13	13–41
T helper CD4+ lymphocyte	1531	410–1590	42	31–60
NK CD16+ lymphocyte	104	90–590	3	5–27
B lymphocyte	1245	90–660	36	6–25
